# An end-to-end sea fog removal network using multiple scattering model

**DOI:** 10.1371/journal.pone.0251337

**Published:** 2021-05-14

**Authors:** Shunmin An, Xixia Huang, Zhangjing Zheng, Linling Wang

**Affiliations:** 1 Institute of Logistics Science and Engineering, Shanghai Maritime University, Shanghai, China; 2 College of Ocean Science and Engineering, Shanghai Maritime University, Shanghai, China; University of Engineering & Technology, Taxila, PAKISTAN

## Abstract

An end-to-end sea fog removal network using multiple scattering model was proposed. In this network, the atmospheric multiple scattering model was re-formulated and used for sea fog removal. Compared with the atmospheric single scattering model, the atmospheric multiple scattering model could more comprehensively consider the effect of multiple scattering, which was important to the dense fog scenes, such as in ocean scene. Therefore, we used the atmospheric multiple scattering model to avoid image blurring. The model can directly generate the dehazing results, and unify the three parameters of the transmission map, the atmospheric light and the blur kernel into one formula. The latest smooth dilation and sub-pixel techniques were used in the network model. The latest techniques can avoid the gridding artifacts and the halo artifacts, the multi-scale sub-network was used to consider the features of multi-scale. In addition, multiple loss functions were used in end-to-end network. In the experimental results, the model was superior to the state-of-the-art models in terms of quantitatively and qualitatively.

## Introduction

In the ocean scene, sea fog is a traditional atmospheric phenomenon. Due to the presence of dense fog in the ocean scene, it may reduce the contrast of the image, thereby affecting many computer vision tasks. Therefore, sea fog removal is a difficult task.

Image dehazing has been extensively studied using the atmospheric single scattering model [1, 2]. This model is mainly used in land scenes or mist scenes, and the image dehazing methods [3–10] are mainly divided into two categories: prior-based method [3, 4, 11–14] and learning-based method [5, 6, 15–22]. The prior-based method uses observations and hypotheses to obtain image prior information, the acquired prior information is used to solve for atmospheric light and transmission map. For example, He et al. [3] proposed the dark channel prior (DCP) [3], and Zhu et al. [4] proposed the color attenuation prior (CAP) [4]. Among them, He et al. [3] found large number of dark channels in the clean images by observing the clean image, so He et al. [3] made an assumption based on this prior and inverted the dehazing image by the atmospheric single scattering model. Prior-based method requires manual extraction of prior knowledge. Among learning-based methods, convolutional neural networks are generally used to extract features [5–9, 18, 19]. For example, Ren et al. [5] proposed single image dehazing via multi-scale convolutional neural networks (MSCNN), which can directly estimate transmission map. Cai et al. [6] recovered the transmission map using convolutional neural network instead of manual extraction of features. It should be noted that all the above methods have a common feature that they use the atmospheric single scattering model to recover dehazing images. The transmission map and the atmospheric light are calculated separately, and without considering multiple scattering. From the application of atmospheric multiple scattering model, the effect of image dehazing using atmospheric multiple scattering model is better than using atmospheric single scattering model. For example, Wang et al. [23] and He et al. [24] used the atmospheric multiple scattering model for image dehazing and had better results compared to using the atmospheric single scattering model. According to the atmospheric scattering physical model proposed by Narasimhan and Nayar [2, 25], the atmospheric single scattering model is a degenerate form of the atmospheric multiple scattering model. The atmospheric single scattering model does not consider multiple scattering. Therefore, the restored images are sub-optimal.

Because of the widespread existence of dense haze and multiple scattering. Many image dehazing methods will be less effective when applied to ocean scenes. To solve this problem, the atmospheric multiple scattering model was proposed and used to remove sea fog, at the same time, the network model and loss function were also proposed. Specifically, the proposed network model was based on reconstructed atmospheric multiple scattering model, which combined multiple parameters into one parameter and estimated to dehazing image. In the end-to-end network, subpixel convolution [26] was used instead of transposed convolution to avoid halo artifacts, and smooth dilation convolution [27] was used instead of transposed convolution to avoid gridding artifacts. In the ocean scene, due to the complexity of detail, structure and texture information, multiple loss functions were proposed to optimize the network, which contained Mean Square Error loss, multi-scale structural similarity loss [28] and perceptual loss [29]. A large number of experiments prove the advantages of this model, compared with the current most advanced model, it performs well in terms of PSNR [30], SSIM [31] and subjective visual quality.

In summary, many dehazing methods do not consider multiple scattering and current research lacks solutions for dehazing under dense fog scenes, such as ocean scene. To overcome these problems, this paper makes the following innovations:

- In order to eliminate the influence of the sea fog, the atmospheric multiple scattering model was reconstructed and used for ocean image dehazing. Specifically, in the reconstructed atmospheric multiple scattering model, the convolution of clean image with blur kernel was simply expressed as Hadamard product, in the reconstructed atmospheric multiple scattering model, the three parameters were fused into one parameter estimate.
- In the end-to-end network, subpixel convolution [26] is used instead of transposed convolution to avoid halo artifacts, and smooth dilation convolution [27] is used instead of transposed convolution to avoid gridding artifacts.
- In the ocean scene, for the complexity of detail, structure and texture information, multiple loss functions were used to optimize the network, which contained Mean Square Error loss, multi-scale structural similarity loss [28], and perceptual loss [29].

The structure of this paper is as follows: In the first section, the existing problems of image dehazing were illustrated. Specifically, explained the challenges of image dehazing in ocean scenes and proposed the solution. In the second section, introduced the relevant work, which mainly included the atmospheric scattering physical model, prior-based methods and learning-based methods. In the third section, the proposed model was analyzed and explained it from the following three aspects: transformed formula, network model and loss function. In the fourth section, qualitative and quantitative experiments have shown the advantages of the method in this paper. In the fifth section, the advantages of the method in this paper were summarized.

## Related work

In the previous section, we mainly summarize the challenges and proposed solutions for image dehazing in the ocean scene. In this section, we will focus on atmospheric scattering physical model, prior-based methods and learning-based methods.

### The atmospheric scattering physical model

The atmospheric scattering physical model [1] can be divided into the atmospheric single scattering model [2] and the atmospheric multiple scattering model [23, 24]. In the applications of the atmospheric multiple scattering model, Wang et al. [23] and He et al. [24] used the atmospheric multiple scattering model to recover the dehazing image, and achieved better effect of dehazing compared with the atmospheric single scattering model. In the ocean scene, the atmospheric multiple scattering model more comprehensively describes and explains the image blur caused by multiple scattering.

#### The atmospheric single scattering model

**Fig 1:**
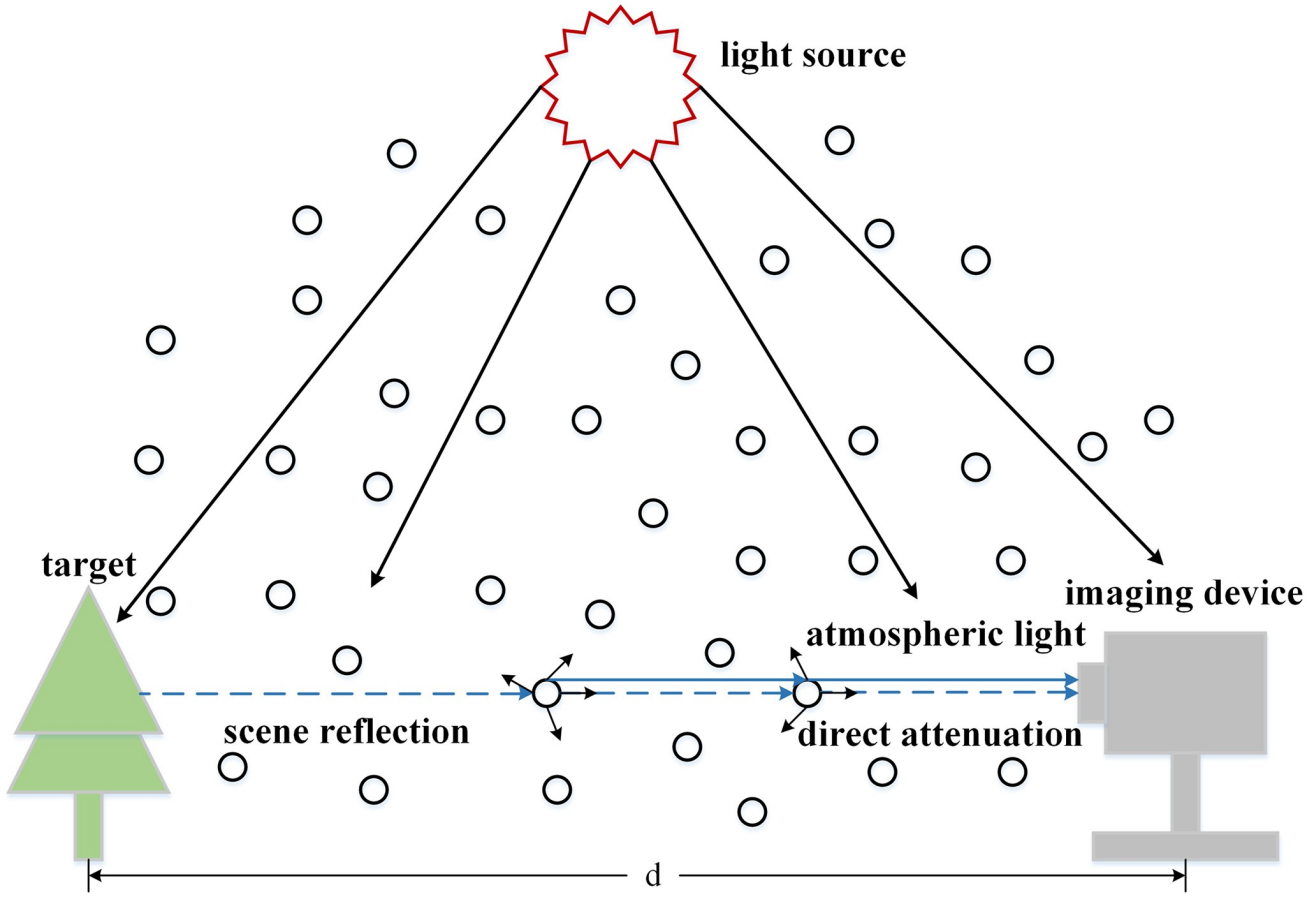
The atmospheric single scattering process, it represents the process of imaging in hazy scenes and only single scattering is considered.

Fig 1 shows the atmospheric single scattering processes, in imaging, the image affected by haze can be represented by the following formula:

I(x)=t(x)⋅J(x)+[1−t(x)]⋅A, (1)

In formula (1), the hazy image and clean image are represented by *I*(*x*) and *J*(*x*), and the atmospheric light is represented as *A*. Where *t*(*x*) is the transmission map, which can be represented as follows:

$$t(x)=e^{-\beta d(x)},$$ (2)

where *β* and *d*(*x*) denote the scattering coefficient and the distance between the target and the imaging device. It should be pointed out that the atmospheric single scattering model ignores multiple scattering in this process.

The atmospheric single scattering model assumes that the reflected light of the scene will be attenuated by the atmospheric particle scattering, and the attenuated part will not interfere with the reflected light of other scenes. Based on the atmospheric single scattering model, when the applied scene fog is thin, this process can be simply viewed as single scattering. However, in the ocean scene, due to the dense haze, the atmospheric single scattering model can’t fully describe the scattering process of reflected light at this time.

#### The atmospheric multiple scattering model

**Fig 2:**
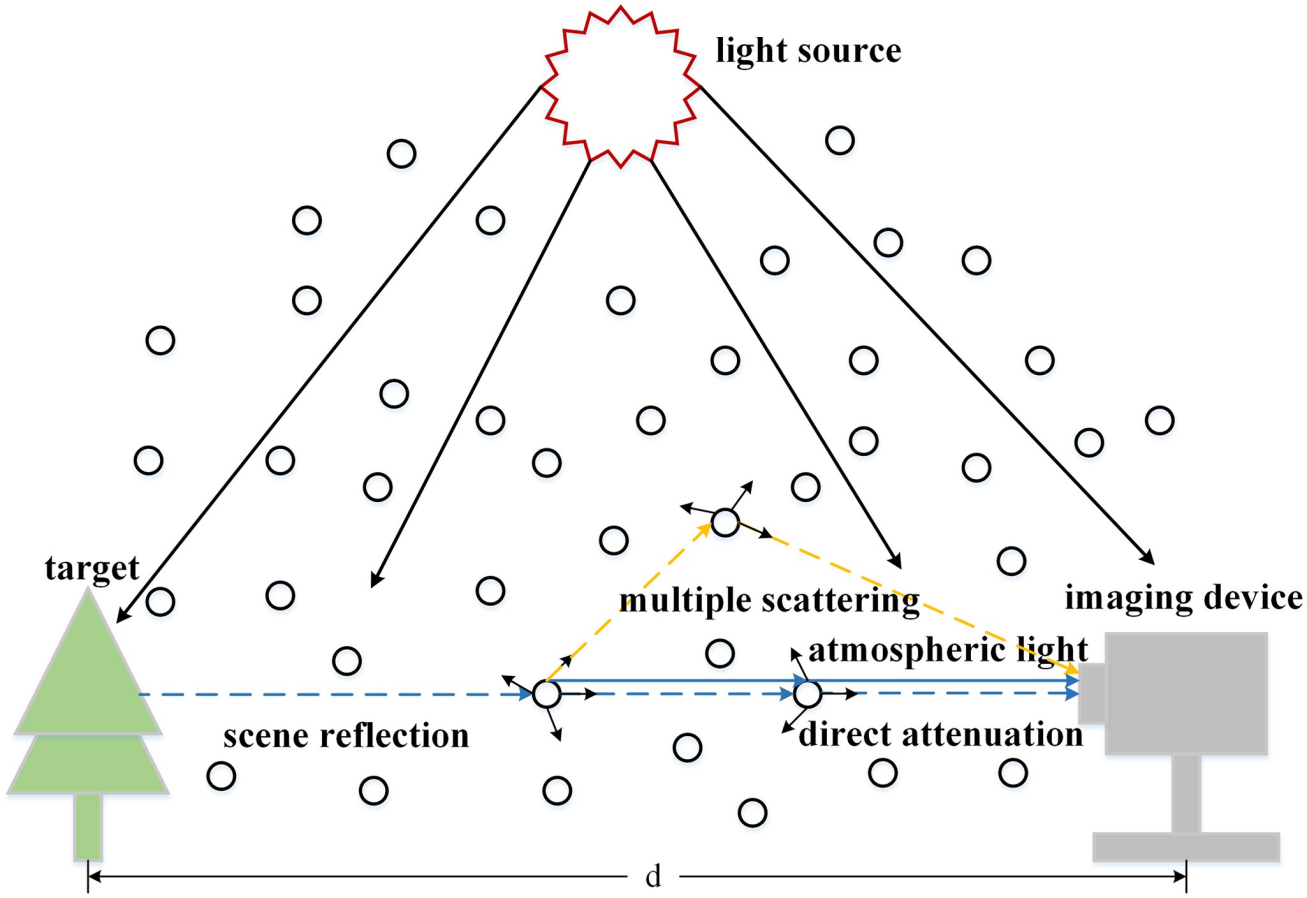
The atmospheric multiple scattering process, it represents the process of imaging in hazy scenes and considers the effect of multiple scattering on the image, it can describe the scattering process more fully.

Fig 2 shows the atmospheric multiple scattering processes. The atmospheric multiple scattering model [32] takes into account the multiple scattering, in the case of multiple scattering, the image affected by haze can be expressed by the following formula:

$$I(x)=[J(x)*h_{A}]\cdot t(x)+A\cdot [1-t(x)],$$ (3)

*h*_*A*_ is the atmospheric point spread function, which is a convolution matrix, and methods of solving this convolution matrix has attracted the interest of researchers, for example, using Monte Carlo and filtering methods [33–35] to solve the convolution matrix. In order to simplify the representation, in the following we denote the convolution matrix *h*_*A*_ by *k* and call it the blur kernel. *is the convolution operator, and *J*(*x*) * *h*_*A*_ denotes the amount of scene radiation affected by multiple scattering effects. According to formula (3), it can be seen that, compared with the atmospheric single scattering model, the atmospheric multiple scattering model considers the influence of multiple scattering on the image.

### Prior-based methods

In image dehazing, there are many prior-based methods of image dehazing [3, 4, 11–14], which restore the dehazing image by using the attributes of the image itself. Through observation and statistics of hazy images, many prior-based methods were proposed [3, 4]. For example, He et al. [3] proposed a dehazing method using dark channel prior (DCP) for image dehazing. Its content is that in outdoor clear images, there are a large number of dark channels in the image, and the value of these dark channels is very small or even close to zero. Based on this observation, the haze-free image was successfully restored. This method is hereafter called DCP [3]. Although the dark channel prior (DCP) method can effectively solve the problem of image dehazing based on observations and assumptions, it relies too much on prior knowledge. It is difficult to achieve desired effect of dehazing when the changing scene is inconsistent with prior knowledge. In addition, Zhu et al. [4] proposed a dehazing method using color attenuation prior (CAP) for image degradation in dehazing, its content is that the haze concentration is proportional to the difference between brightness and saturation. Based on this observation, this method models the scene depth and restores the information of scene depth, and uses the established model to dehaze. Although the color attenuation prior (CAP) can effectively solve the problem of image degradation in image dehazing, it relies on statistical information and cannot be completely dehazing in many scenes. Among the above prior-based methods, although the prior-based methods have made great progress in image dehazing, they still rely on various prior knowledge and have certain limitations.

### Learning-based methods

In order to avoid depending on prior knowledge, many dehazing methods use neural networks instead of manually extracting features of haze, and they are able to avoid the reliance on prior knowledge very well. Therefore, many dehazing methods using convolutional neural network have been proposed [5, 6, 15–22]. For example, Ren et al. [5] proposed an image dehazing method based on multi-scale convolutional neural network to solve the problem of over-reliance on prior knowledge in the prior-based dehazing method. Ren et al. [5] estimated the transmission map through the multi-scale convolutional neural network and finally realized image dehazing. Although the multi-scale convolutional neural network can restore the transmission map, it estimates the transmission map and atmospheric light separately, which will result in the accumulation of errors and cannot effectively remove haze. In addition, Li et al. [15] addressed the problem that separate estimation of transmission map and atmospheric light would cause error accumulation, which fused transmission map and atmospheric light into one parameter so only one parameter needs to be estimated, thus successfully avoided the problem of error accumulation, and restored the haze-free image through the end-to-end convolutional neural network. This method is hereafter called AOD-Net [15]. Although it avoids the accumulation of errors to a certain extent by fusing the transmission map and the atmospheric light into one parameter, the estimation of the parameters is not always accurate with a shallow convolutional neural network, and there is still some haze in the image after dehazing. In summary, although learning-based methods have achieved good results in image dehazing, they are usually based on the atmospheric single scattering physical model, which do not consider the effect of multiple scattering on the image.

Although both AOD-Net [15] and the method of this paper both fuse multiple parameters into a single parameter. Compared to AOD-Net [15], the method of this paper is different in the physical model, network structure, and loss function. Specifically, first, in terms of physical model, AOD-Net [15] uses the atmospheric single scattering physical model, which is not a dehazing method designed for oceanic scenes. Instead, the method of this paper uses the atmospheric multiple scattering physical model, which can avoid the blurring of images caused by multiple scattering, especially in the ocean scene. Second, the method of this paper uses a new network structure, which uses the latest smooth dilation [27] and sub-pixel [26] techniques to avoid gridding artifacts and the halo artifacts, and uses multi-scale sub-network to fuse multi-scale feature information, while the AOD-Net method only uses a spanning-connected convolutional neural network as the network model. Third, the loss function is different, we proposed and used multiple loss functions to optimize the network model, specifically, the method of this paper use Mean Square Error loss, multi-scale structural similarity loss [28], and perceptual loss [29], which can not only help the network focus on image details, but also consider the texture and structural information of the image during the training process. In contrast, AOD-Net only uses Mean Square Error loss.

## Method

In this section, an end-to-end sea fog removal network using multiple scattering model was introduced. Firstly, the atmospheric multiple scattering model was reconstructed, and the obtained atmospheric multiple scattering model could be optimized by fusing multiple parameters into one parameter. On this basis, the network model was designed, in order to enable the network to fully learn the characteristics of haze and accurately estimate parameters, we used the latest smooth dilation [27] and sub-pixel [26] techniques, as well as multi-scale subnetwork. Finally, multiple loss functions were introduced. Specifically, we used Mean Square Error loss, multi-scale structural similarity loss [28] and perceptual loss [29], which not only help the network pay attention to the details of the image, but also consider the texture and structure information training of the image during training. In this paper, the dataset can be found at: https://doi.org/10.17605/OSF.IO/5VHSN. It is important to note that this data set originates as part of RESIDE data set.

### Transformed formula

**Fig 3:**
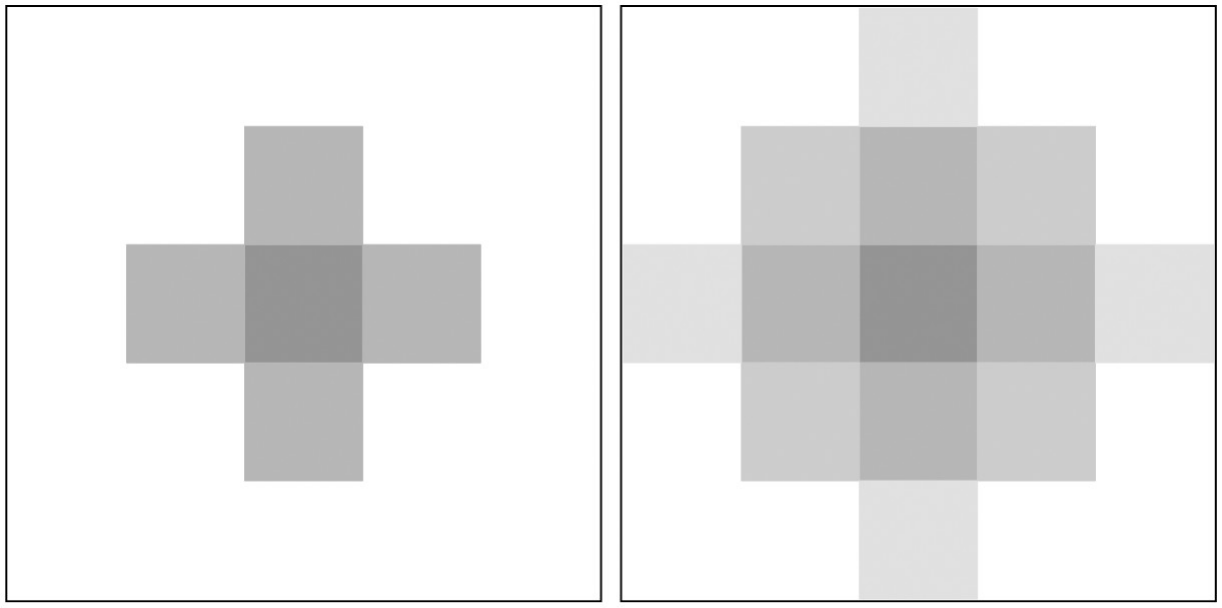
The dispersion spot from multiple scattering, due to the existence of multiple scattering, the clear image will appear blur phenomenon, which is affected by the dispersion spot formed by multiple scattering.

In the ocean scene, if multiple scattering is not taken into account, halo and blur will often appear in the dehazing results. The light is scattered many times and reaches the camera at different angles, and finally forms the dispersion spot on the imaging plane. As depicted in Fig 3, the dispersion spot is caused by multiple scattering, there will be halo and blur in the result of the image. It is for this reason that many image dehazing methods applied to ocean scene will result in halo and blur.

By the atmospheric scattering model in (3), the image under the influence of multiple scattering is obtained by

$$J(x)*k=\frac{1}{t(x)}I(x)-A\frac{1}{t(x)}+A.$$ (4)

As revealed by Fig 3, it takes into account the effects of multiple scattering, so that the image *J*(*x*) * *k* under the influence of multiple scattering will appear halo and blur. Especially in the ocean scene, due to dense haze and small targets, multiple scattering has a very serious impact on the image. In many dehazing methods, most of them mainly consider the land scene, where the haze is thin and the effect of multiple scattering on the image is not very serious, so they do not consider the effect of dispersion spots, and according to formula (4) we can easily see that in the ocean scene, the result we get *J*(*x*) * *k* is suboptimal because the effect of multiple scattering is not considered. Therefore, further optimization is important. However, deconvolution and the estimation of blur kernel is a challenging problem in image processing.

Based on the above factors, blurring is widespread in hazy images, therefore, referring to formula (4), the image under the influence of multiple scattering is obtained by convolution of the blur kernel and clean image. However, estimating the blur kernel and deconvolution are difficult. In the method of image deblurring and super-resolution, a number of researchers have proposed methods of estimating blur kernel and deconvolution. For example, S. Metari et al. [33] viewed the blur kernel generated by multiple scattering as Gaussian blur kernel and used Gaussian filter to filter the images to simulate various weather conditions. Gu et al. [32] used the SFT layer to view the convolution of low-resolution image with blur kernel as Hadamard product in the same space, and finally succeeded in recovering high-resolution image. Since blurring affects the whole image, according to the literature [32] the convolution of the blur kernel and the image can be seen as Hadamard product in spatial consistency, thus the blur kernel and the clean image can be seen to be spatially consistent, and inspired by the literature [32], we express the convolution of the clean image with the blur kernel simply as Hadamard product. To this end, the formula in (4) is re-expressed as:

$$J(x)\cdot k=\frac{1}{t(x)}I(x)-A\frac{1}{t(x)}+A.$$ (5)

As explained in the second section, previous methods usually estimate *t*(*x*) and *A* separately. They estimate multiple parameters, and this optimization leads to sub-optimal solution, in the atmospheric multiple scattering model, estimating the three parameters separately will result in the accumulation of errors and the estimation of the blur kernel is more difficult. Therefore, we fused the three parameters into a single parameter *K*(*x*) and only estimated this one parameter. To this end, the formula in (5) is re-expressed as:

$$\begin{array}{l} J(x)=K(x)I(x)-K(x)+1,where\\ K(x)=\frac{\frac{1}{t(x)}(I(x)-A)+(A-k)}{k(I(x)-1)}. \end{array}$$ (6)

Three parameters were fused into one parameter based on the atmospheric multiple scattering model. The unique parameter estimation will affect the dehazing effect, and we need to build a deep network model that can learn the features and estimate the parameters accurately.

### Network design

**Fig 4:**
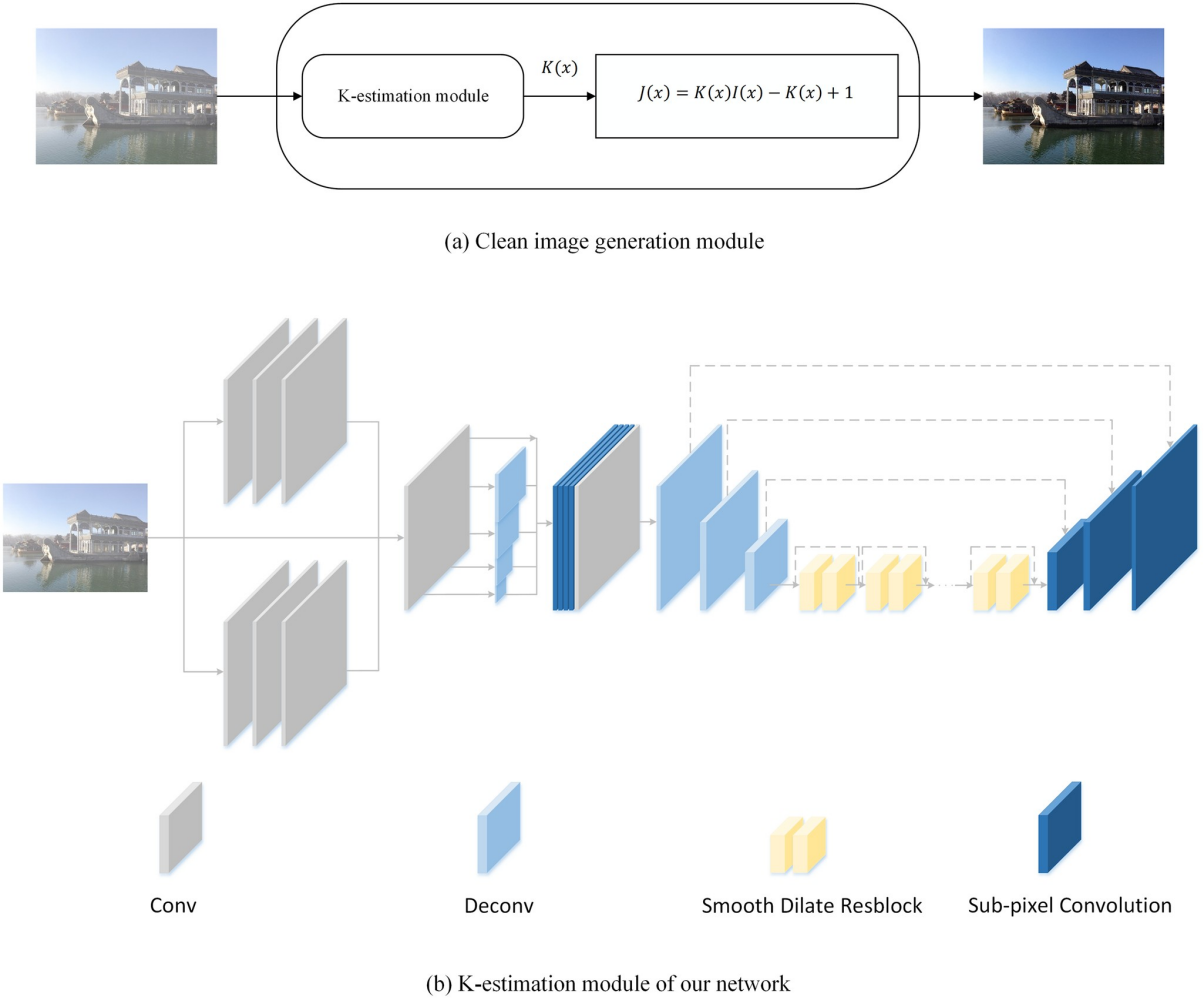
The end-to-end network, it is composed of two parts, the K-estimation module is used to estimate parameters, and the clean image generation module uses the re-formulated atmospheric multiple scattering model to generate clean images.

The designed network needs to learn the characteristics of hazy images and estimate parameters accurately. This network can input hazy images and directly output clean images [36], the designed network model can accomplish haze feature extraction and accurate estimation of parameters. The network model consists of two parts, according to Fig 4, it can be seen that the K-estimation module was used to estimate the parameter, and the clean image generation module was used to recover the dehazing images, the whole network was implemented in an end-to-end manner.

As depicted in Fig 4(b), in the K-estimation module, the latest smooth dilation [27] and sub-pixel [26] techniques are used, and multi-scale subnetwork is used to fuse multi-scale features. Specifically, first, we used convolution kernels of different sizes to convolve the input hazy image, in which the neural network can learn the features of haze under different receptive fields. After this, the feature maps were input into multi-scale sub-network, which can help the neural network to learn the features of haze at different scales. Finally, to enhance the learning ability and dense prediction ability of the neural network, the latest smooth dilation [27] and sub-pixel [26] techniques were used, which can improve the accuracy of the network prediction and ensure that the neural network recovers the information of dense prediction.

**Fig 5:**
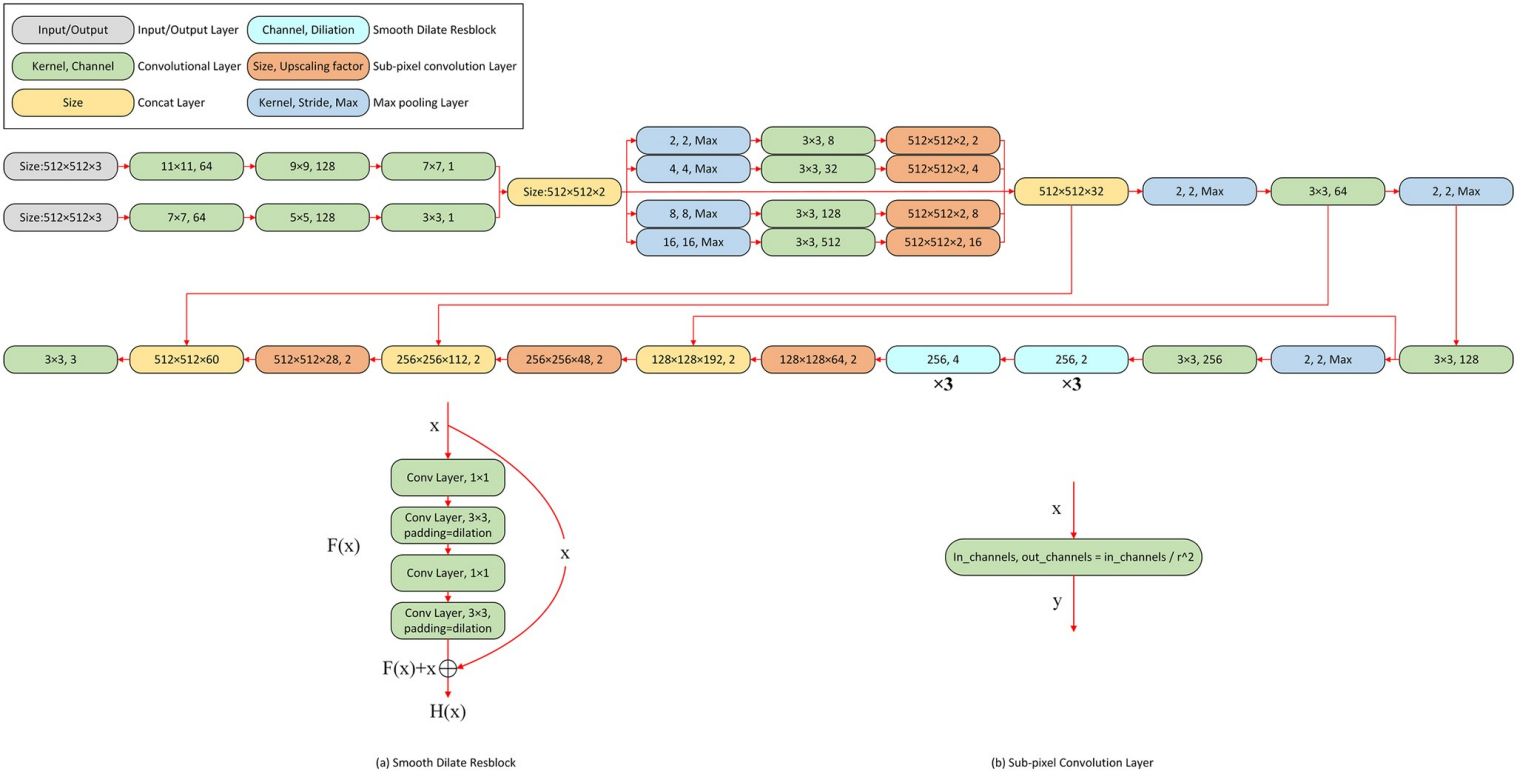
A more detailed network structure is presented, which consists of convolutional layer, max pooling layer, sub-pixel convolutional layer, smooth dilate resblock and concat layer.

In this process, the learning ability and prediction accuracy of the network model are directly related to the effectiveness of dehazing. Since image dehazing is an intensive and complex prediction task, the use of smooth dilation convolution [27], sub-pixel convolution [26] and multi-scale sub-network can improve network performance and avoid information loss. Prediction of parameter and learning of characteristics of haze are very important. Therefore, it is necessary to increase the depth of the network model, in order to solve the above problem, smooth dilation convolution was used in this network model, unlike the dilation convolution which often cause the gridding artifacts, smooth dilation convolution can avoid this problem, which can increase the depth of the network and enhance the reception field of the network. In addition, the deconvolution process will cause information loss, which will affect the learning of haze feature and the estimation of parameter. Therefore, sub-pixel convolution [26] was used, it avoided the loss of information during deconvolution process and increased the depth of the network and the receiving field. Finally, in order to improve the depth and learning ability of the network, multi-scale sub-network was used to enhance the performance of the network, which can help the network model to learn multi-scale information. Fig 5 shows a more specific network structure, and in the following sections, the important parts of the network structure are described in detail.

#### Multi-scale sub-network

**Fig 6:**
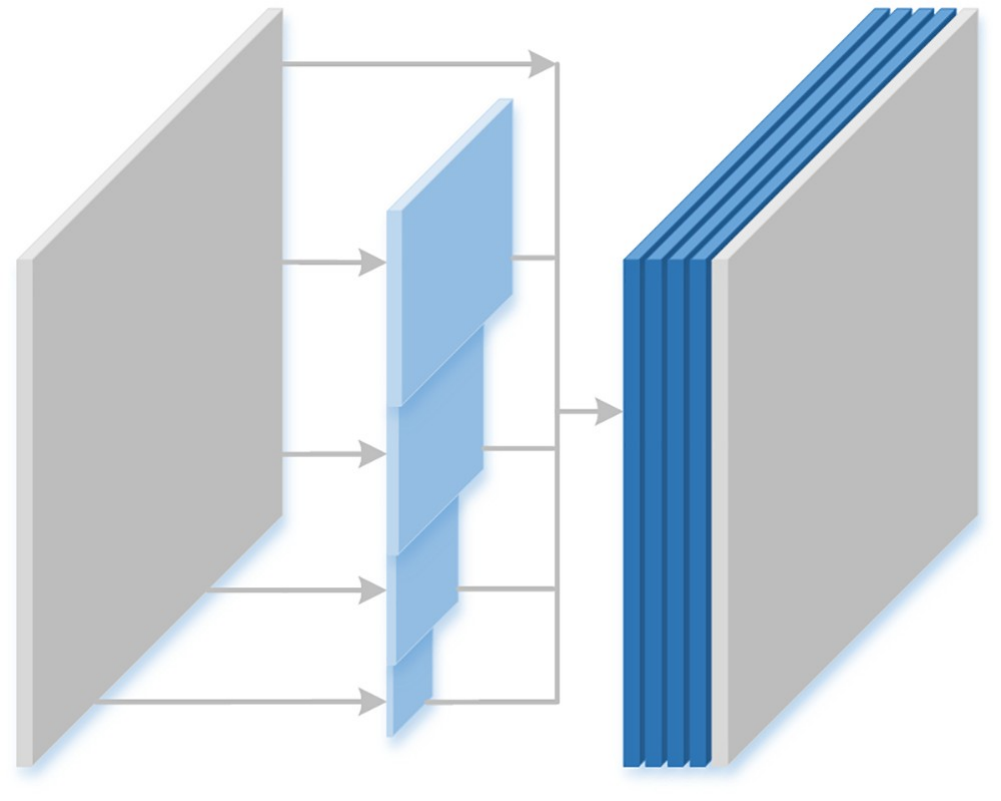
Multi-scale operation, it down-samples the input feature maps by 1/2, 1/4, 1/8, and 1/16, and then restores them to the original size connection by sub-pixel convolution.

In network model, the fusion of features at different levels tends to improve the performance of the model [37, 38]. Since image dehazing is an intensive prediction task, multi-scale sub-network was used in the network model, which can help neural network to learn multi-scale information. Specifically, first, feature maps of different scales were obtained through down-sampling operations, and it was able to learn feature information of haze at different scales, then connect the feature maps by sub-pixel convolution [26]. In this process, the multi-scale sub-network integrated the characteristic information of haze at different scales, which can help the neural network to better learn the characteristic information of haze. Fig 6 shows the multi-scale operation.

#### Smooth dilation convolution

In neural networks, successive down-sampling layers will degrade the resolution. Therefore, in the tasks of target detection and semantic segmentation, the dilated convolution [27] can obtain a larger receptive field, which results in more dense data, it was able to preserve the spatial features of the image very well without loss of image information. However, dilated convolution may lead to loss of spatial continuous information and appearance of grid artifacts. To alleviate it, Yu et al. [39] proposed to increase the interaction between input units by adding an additional convolutional layer. In the network, we insert six smooth dilated convolution residual blocks and call them “Smooth Dilated Resblock”.

#### Sub-pixel convolution

In neural network, successive transposed convolution operations cannot fully recover low-resolution image. Therefore, for intensive recovery tasks like super-resolution, the up-sampling process of the transposed convolution is sub-optimal and increases computational complexity. To solve this problem, Shi et al. [26] proposed a new sub-pixel convolution to replace the transposed convolution.

Sub-pixel convolution [26] inputs *H* × *W* low-resolution image and converts them into *rH* × *rW* high-resolution image by sub-pixel operation. The process is to obtain feature maps of *r*^2^ channels through convolution, then obtain high-resolution image through regular reorganization.

### Loss function design

At present, many learning-based image dehazing methods [6, 15, 16] only use Mean Square Error loss. Although they can recover the original image from the hazy image, the mean square error loss cannot fully express the image that the human visual system intuitively perceives due to there are complex details, structure and texture in ocean scenes. To efficiently address this issue, multiple loss functions were used to optimize the network, it includes Mean Square Error loss, multi-scale structural similarity loss [28] and perceptual loss [29].

#### Multi-Scale structural similarity loss

The MS_SSIM [28] for two images *x*, *y* is defined as:

$$\begin{array}{l} MS\_SSIM(x,y)=\prod_{j=1}^{M}SSIM(x_{j},y_{j}),where\\ SSIM(x,y)=\frac{\left(2\mu_{x}\mu_{y}+C_{1})(2\sigma_{xy}+C_{2}\right)}{\left(\mu_{x}^{2}+\mu_{y}^{2}+C_{1})(\sigma_{x}^{2}+\sigma_{y}^{2}+C_{2}\right)}. \end{array}$$ (7)

Where *μ* and *σ* denote the means and standard deviation of the image. The higher the similarity of two images, then the higher the MS_SSIM, for two identical images, MS_SSIM [28] is equal to one. The loss function of MS_SSIM can be written as follows:

$$L_{MS\_SSIM}=-MS\_SSIM(x,y).$$ (8)

#### Perceptual loss

In the perceptual loss [29] function, the loss network was obtained by the pre-trained model, input clean image and dehazing result, and minimize loss between feature maps, which can make the high-level information closer.

$$L_{feat}^{\phi ,j}(\hat{y},y)=\frac{1}{C_{j}H_{j}W_{j}}{\left\|\phi_{j}(\hat{y})-\phi_{j}(y)\right\|}_{2}^{2},$$ (9)

where *j* and *ϕ* denote the layer *j* of the network and the loss network respectively, the loss obtained will be the square-normalized euclidian distance.

In this paper, multiple loss functions were applied. As shown in formula (10), where *α* and *β* are the positive weights of the corresponding loss functions:

$$L=MSE+\alpha L_{MS\_SSIM}+\beta L_{feat}^{\phi ,j}(\hat{y},y).$$ (10)

## Experiments

In this section, a large number of experiments were carried out to verify the validity of the model. Specifically, the dehazing results of the proposed method were verified on the synthetic data set and the real data set. In addition, this paper also compared five state-of-the-art dehazing methods. The experimental results show that the method of this paper performs well in the qualitative and quantitative comparisons.

The training set of the model consists of 13580 synthetic hazy images of oceans and lakes. Similarly, the test data set consists of 1575 synthetic haze images of oceans and lakes. In this paper, the dataset can be found at: https://doi.org/10.17605/OSF.IO/5VHSN. It includes the training set and the synthetic image test set. In addition, the images used for the real-world image comparison experiment were obtained from the Internet, real-world images are available at: https://doi.org/10.17605/OSF.IO/N2BDG. By default, the optimizer and learning rate are Adam and 0.0001, and the entire network is trained for 10 epochs.

### Quantitative results on synthetic images

**Fig 7:**
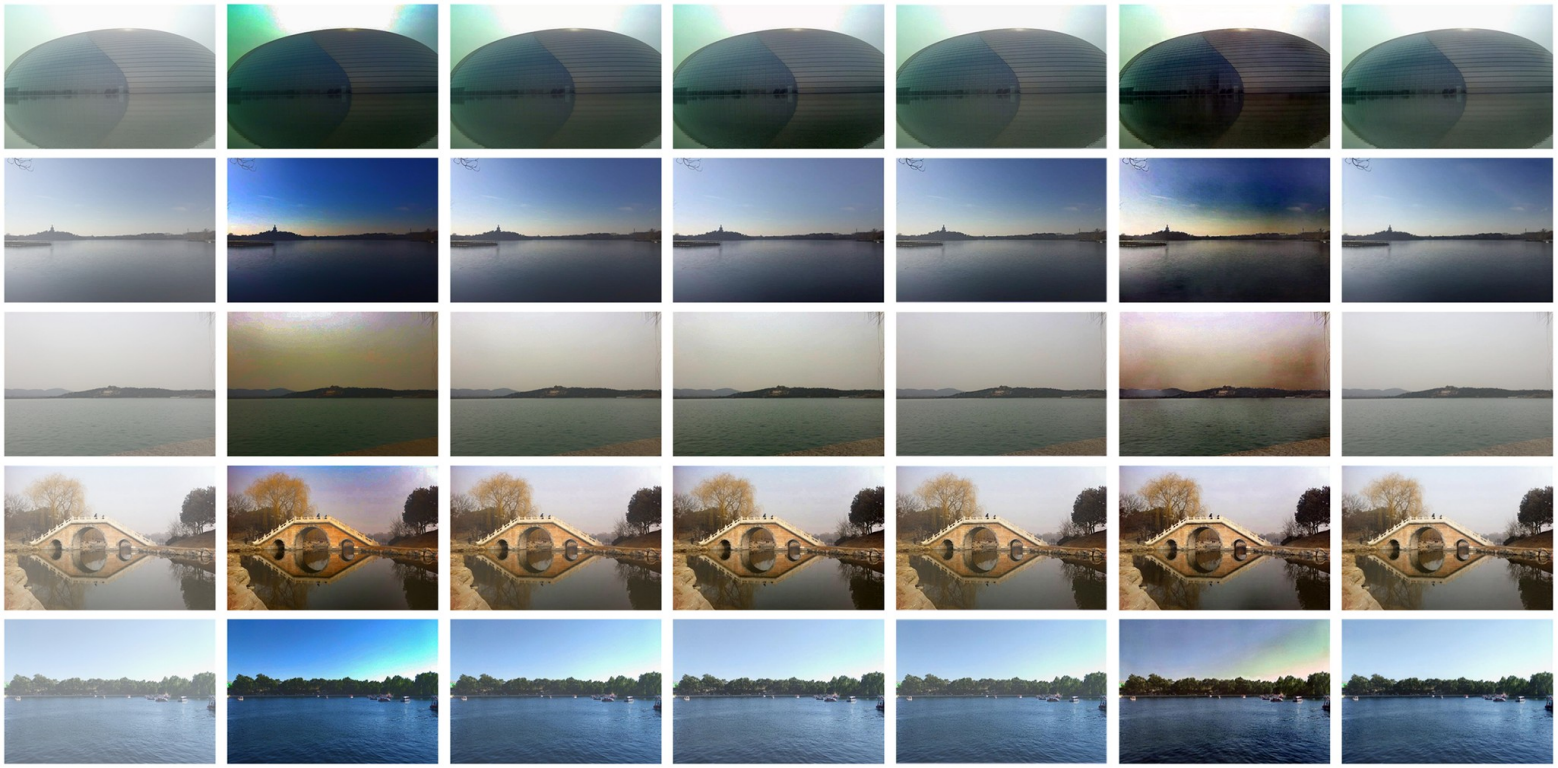
Dehazed results of synthetic images, we compared five methods of dehazing, here shows the effect of some pictures.

In the comparative experiment of synthetic images, dehazing method of this paper was compared with five state-of-the-art dehazing methods. Specifically, we selected two prior-based methods and three learning-based methods, which ensured that the results of the comparison experiments could be analyzed from different perspectives, and the five dehazing methods are as follows: DCP [3], CAP [4], DehazeNet [6], AOD-Net [15] and GCAN [18]. In terms of evaluation indexes [40, 41], PSNR [30] and SSIM [31] were used for quantitative evaluation. Fig 7 shows the dehazing results of synthetic images of different scenes under the six methods. Table 1 shows the PSNR and SSIM results for the dehazing results in Fig 7.

**Table 1:** Quantitative SSIM and PSNR results on the image dehazing, on test data set.

Metrics	DCP [3]	CAP [4]	DehazeNet [6]	AOD-Net [15]	GCAN [18]	Our result
PSNR	18.24	20.78	21.26	22.38	18.16	24.12
SSIM	0.84	0.88	0.87	0.91	0.83	0.93

Fig 7 displays five synthetic hazy images of ocean and lake scenes and their dehazing results. Through observation, it can be found that DCP [3] and CAP [4] darken the brightness of the dehazing result. For DehazeNet [6] and AOD-Net [15], they usually cannot completely eliminate haze. For GCAN [18], we find that it has color distortion and halo. It can be observed from Fig 7 that our method of this paper is more effective compared with the five dehazing methods. It can preserve edge details well, and will not show halo and blur.

As shown in Table 1, We compared the proposed model with five state-of-the-art dehazing methods: DCP [3], CAP [4], DehazeNet [6], AOD-Net [15] and GCAN [18]. According to Table 1, it can be concluded that the proposed method has higher PSNR and SSIM indexes than dehazing methods using atmospheric single scattering model, which can indicate that the reconstructed atmospheric multiple scattering model and network model are capable of the task of ocean scenes, and the dehazing effect is very good.

### Qualitative visual results on real-world images

In the comparison experiment of real images, in order to show the difference between dehazing method of this paper and others using atmospheric single scattering model, five advanced dehazing methods were used in the comparative experiment, and it should be noted that they all use the atmospheric single scattering model. We have selected three sea fog images on the Internet, and they are all from real ocean scenes with hazy images. The five dehazing methods were as follows: DCP [3], CAP [4], DehazeNet [6], AOD-Net [15] and GCAN [18].

**Fig 8:**
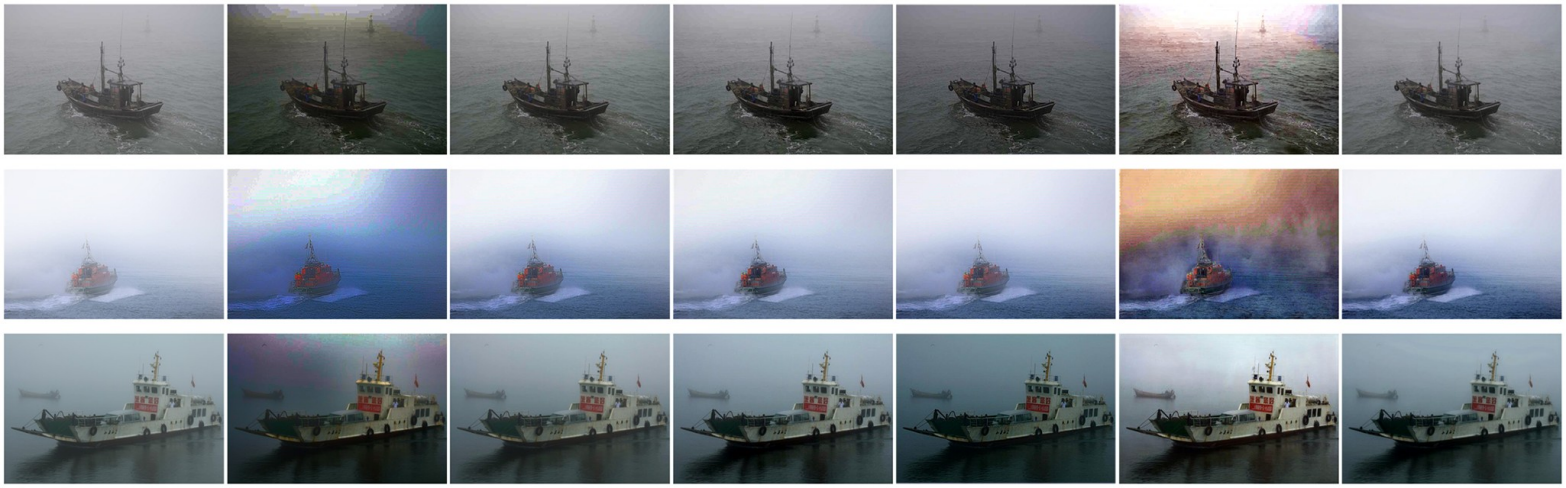
Dehazed results of real-world images, we compared five methods of dehazing, here shows the effect of some pictures.

Fig 8 shows the dehazing results in a real ocean scene. According to the observation, it could be found that halo and blur appeared in the dehazing results of DCP [3] and GCAN [18], their visual effects were not good, and the details could not be restored well. For CAP [4], DehazeNet [6] and AOD-Net [15], their edge details and structural information cannot be well represented, and there was blur around the edges of the object. In the ocean scene, the method of this paper restored image detail, structure and texture while eliminating sea haze to the greatest extent. Therefore, the method of this paper is good at removing sea fog in the ocean scene.

## Conclusion

In this paper, an end-to-end sea fog removal network using multiple scattering model was proposed, the end-to-end network is based on reformulated atmospheric multiple scattering model, and use multiple loss functions for sea fog removal. In addition, the latest sub-pixel and smooth dilated techniques were used in the network. They can not only enhance the learning ability and predictive ability of network, but also avoid gridding artifacts and halo artifacts. Finally, multiple loss functions were used to constrain the network structure, which can take into account the image details and pay attention to the texture and structure of the image. The experiment shows the effectiveness and significance of the method.
